# The Importance of Ionic Liquids in the Modification of Starch and Processing of Starch-Based Materials

**DOI:** 10.3390/ma13204479

**Published:** 2020-10-09

**Authors:** Sylwia Ptak, Arkadiusz Zarski, Janusz Kapusniak

**Affiliations:** Department of Dietetics and Food Studies, Jan Dlugosz University in Czestochowa, Armii Krajowej 13/15 ave., 42-200 Czestochowa, Poland; s.ptak@ujd.edu.pl (S.P.); arkadiusz.zarski@ajd.czest.pl (A.Z.)

**Keywords:** ionic liquids, starch modification, solvent, catalyst, plasticizer, compatibilizer, biocatalysis in ionic liquids

## Abstract

The main applications of ionic liquids in chemistry and material research on one of the most important natural polymers—starch—are presented in this review. A brief characterization of ionic liquids and the advantages and disadvantages of using them in the modification and processing of polysaccharides is presented. The latest reports on the use of various ionic liquids as solvents or co-solvents; as media for synthesizing starch derivatives in oxidation, etherification, esterification, and transesterification, with particular emphasis on biocatalyzed reactions; and as plasticizers or compatibilizers in the processing of starch-based polymers have been investigated. The current trends, possibilities, and limitations of using this type of compound for the production of functional starch-based materials are presented.

## 1. Introduction

One of the main problems in the modern synthesis or modification of various types of materials is the use of solvents and reaction media that generate a number of threats, particularly environmental ones. These threats result from the source and synthesis of the solvent, its functional properties, and, finally, its disposal [1]. This has led to the trend of designing and using pro-ecological solvents. Solvents that have proven successful in this role are water [2,3], ionic liquids (ILs) [4,5,6], supercritical fluids [7,8,9], liquid polymers [10,11], and solvents derived directly from biomass [12]. Even if a classic solvent is replaced with a green substitute, the reaction may turn out to be less efficient, generating more byproducts or requiring additional raw materials and energy. Knowing whether a solvent or solvent system can provide a more balanced reaction/method than others is crucial to determine whether the total benefit outweighs the losses. ILs are able to dissolve inorganic, organic, and organometallic compounds (catalysts) and polymers [13,14]. Most reports on the possibility of carrying out modifications of carbohydrate polymers and using them as reaction media or solvents have appeared in the last 20 years. The interest in the use of ILs is growing due to their unique physicochemical properties, such as non-flammability, low vapor pressure, low melting point, and high recyclability. They were even considered as attractive substitutes for classic organic solvents or other catalysts (Table 1). Demand for ILs is growing due to their unique properties, wide range of practical applications, and commercial potential. In 2015, the global ILs market was valued at USD 20.4 million; however, it is estimated that, in 2021, the market size of ILs will reach the value of USD 39.6 million [15]. There are many studies on the use of ILs in chemistry and the processing of the main carbohydrate polymers, especially cellulose and chitosan. Therefore, full attention is paid to the third most important natural polymer—starch—in this review. One of the first applications of ILs for the modification of starch was to synthesize its acetates in the 1-butyl-3-methylimidazolium chloride environment, further catalyzed by pyridine. So far, the mentioned chloride has been the most commonly used liquid in uncatalyzed as well as chemically or enzymatically catalyzed modification of starch. ILs and other solvents are used in starch modification to loosen its macrostructure by disrupting intramolecular bonds, thus increasing its reactivity [16]. To shorten the time of the partial or complete gelatinization/solubilization of starch, reactions in ILs can be carried out with other modifications or techniques such as microwave radiation. Over the last few years, literature reports have shown that efficient biocatalysis can be also performed with the use of these liquids [17]. Of all the previously synthesized and well-characterized ILs, the most common ones used to modify polysaccharides are those with an imidazolium cation structure and a simple inorganic anion, especially halide. Studies on dissolution and gelatinization in ILs have focused on starches of various botanical origins but only in the synthesis of their carbonyl derivatives, mainly from corn, potato, or cassava [18]. In many cases, the use of certain ILs results in the efficient production of functional starch derivatives under ecofriendly conditions, considering the green chemistry and sustainable development trend, which is presented in this review. Special emphasis was placed on the possibility of using ILs in biocatalyzed reactions.

## 2. ILs as “Green” Designing Compounds

The first salt of this group was synthesized by Ray and Rakshit in 1911 [20]. These were relatively unstable ethylammonium, di- and tri-methylammonium nitrates. A few years later, Walden successfully synthesized the stable protic ionic liquid by reacting ethylamine with concentrated nitric acid [21]. The research interest in these compounds returned in 1990; it was found that salts chemically stable at room temperature, composed mainly of such anions as BF_4_^−^, NO_3_, PF_6_^−^, SO_4_^2−^, and imidazolium or pyrimidinium cations, could also be synthesized [22]. The term used to describe such compounds was first adopted in 1996. Previously, other names, such as molten salts, non-aqueous ionic liquids, or liquid organic salts, were used. ILs are compounds with an ionic structure that consists of an organic cation and organic or inorganic anion. Their melting points are lower than the boiling point of water [23]. Usually, the temperatures, which are relatively different, are between −20 and 100 °C. Ions in these compounds are characterized by their large ionic radius and a low degree of symmetry. Mainly, the cation exhibits an asymmetrical character which depends on the presence of alkyl substituents. In addition, cations are arranged in a larger volume, and the charges of these ions can be distributed among several atoms through the phenomenon of resonance, which strengthens the effect of the energy drop in the crystal lattice. This results in a decrease in the melting point of the ILs [24]. The anions in these compounds are usually much smaller.

**Figure 1:**
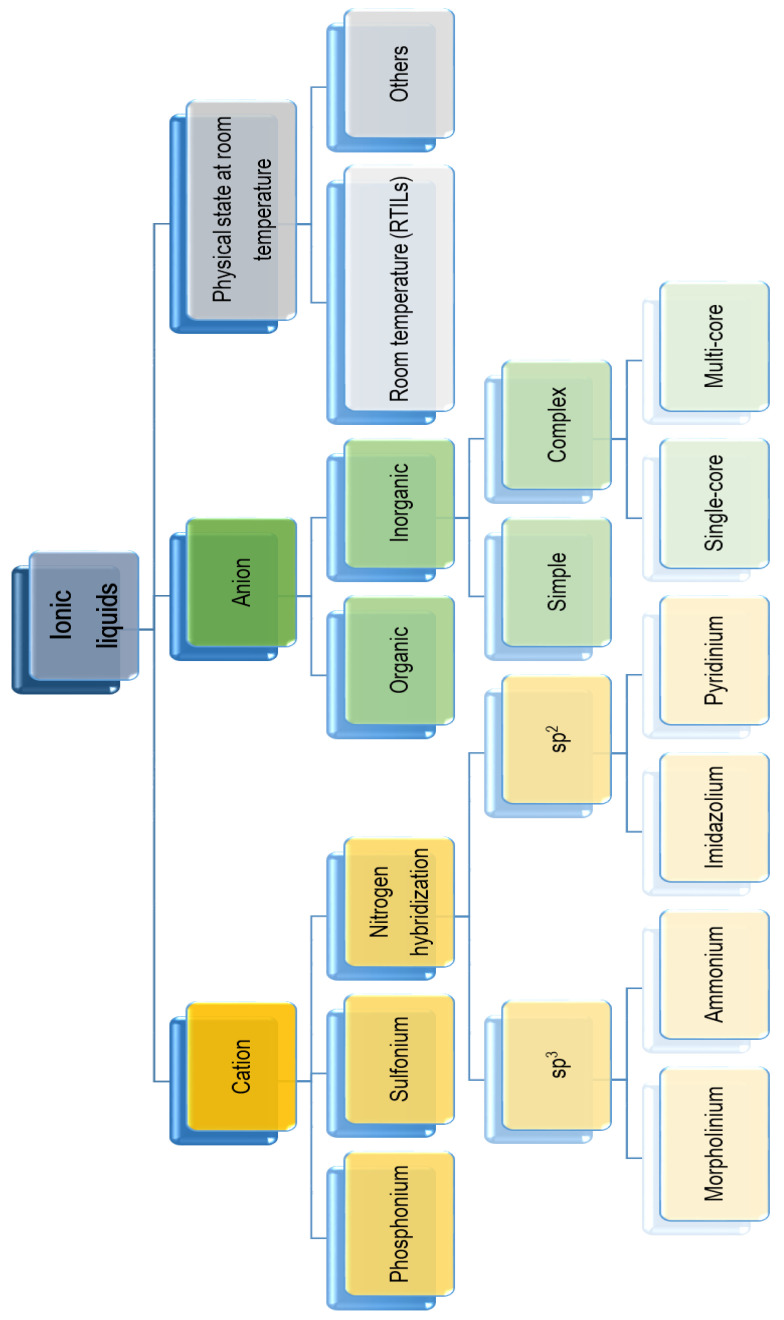
The general classification of ionic liquids [25].

Based on the structure of the cation, the following liquids can be identified and distinguished: sulfonium, phosphonium, morpholinium, ammonium (with sp^2^ nitrogen hybridization), pyridinium, and imidazolium (with sp^3^ nitrogen hybridization) (Figure 1).

**Figure 2:**
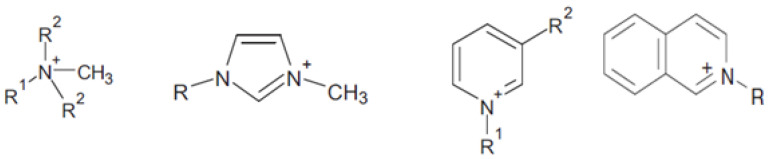
Examples of cations most often included in the structure of ILs.

In turn, based on the anion structure, they can be categorized as ILs with an organic or inorganic anion, and the latter as compounds with a simple inorganic or complex single or dual-core anion. Another method of division takes into account the symmetry of the compound (symmetrical or asymmetrical), as well as the presence of an acid proton (protic or aprotic) [26,27]. Among the cations, the most common are ammonium, pyridinium, and imidazolium (Figure 2); among the anions, the most common are Cl^−^, I^−^, Br^−^, NO_3_^−^, AlCl_4_^−^, PF_6_^−^, BF_4_^−^, and, less often, organic anions such as acetate, benzoate, lactate, and salicylate [28]. ILs are considered as “design solvents” because there may be up to 10^18^ cation–anion connections, and the specificity of such a connection makes it quite easy to control the properties of the newly formed liquids. The physicochemical properties largely depend on the structure of the cation and the type of anion [29,30,31,32]. Additionally, ILs can be modified by mutual mixing or mixing with conventional solvents. Moreover, simple modifications of the structures of ILs can lead to profound changes in their properties, which gives them a great advantage over classic solvents. Most notably, they are non-volatile in the liquid state due to their low vapor pressure. Therefore, it can be stated that they are environmentally friendly compounds, i.e., they can be classified as green solvents [33]. However, this is a moot point due to their low biodegradability, biocompatibility, and sustainability [34]. Another advantage of ILs is their high thermal stability (decomposition temperature in the range of 300–350 °C and thermal indifference even at reduced pressures and increased temperatures). Most of these compounds are characterized by strong polarity [23]. Moreover, they are distinguishable by their high electrical conductivity and wide electrochemical window. Properties of ILs, such as the melting point, density, viscosity, or even surface activity, can be easily controlled by structural interference. The melting point of an IL depends, to a large extent, on the length of the alkyl substituent, its degree of branching, and the kind of anion present [19,23]. The density, in turn, depends on the structure of this substituent itself—the more carbon atoms in it, the lower its density. The viscosity of the IL mainly depends on the type of anion, changes in temperature, and presence of water or various organic solvents and inorganic salts. In turn, its surface activity is mostly influenced by the structure of the cation [35,36].

One of the most important disadvantages of ionic liquids is difficulty with recovering them. Any contamination of ionic liquids has a great influence on their physical and chemical properties. The most common impurities are halides, water, unreacted substrates, and colored impurities—products of oxidation of the substrates. Due to the very low vapor pressure of ionic liquids, traditional purification by distillation is impossible. This property, however, can be used to distill any volatile impurities from ionic liquids. The best way to ensure a high-quality product is to thoroughly purify the substrates used in the synthesis of ionic liquids [37,38]. The most common and most abundant pollutant is water. Other solvents can be almost completely removed by heating the ionic liquid under reduced pressure for several hours. Water is the most difficult solvent for its removal, even from ionic liquids that exhibit high hydrophobicity. Another, often very troublesome, pollutant is halide ions. These impurities may limit the use of ionic liquids as solvents, because there is a risk of deactivating the metal catalysts or disturbing the course of the reactions. Unfortunately, there is no universal method for purifying ILs, mainly due to the diversity of their structures. There are, however, several strategies to minimize the halide ion content [35,39,40].

Despite the difficulties with recycling, ionic liquids have a great advantage over classic solvents. Ionic liquids facilitate separation of the end product and the recycling of used catalysts, especially in situations where the reaction medium is a two-phase system. Another feature, thanks to which ionic liquids gain an advantage over conventional organic solvents, is the high degree of conversion and negligible losses of ionic solvent and catalyst, which guarantees their repeated use without loss of activity or durability. Ionic liquids enable an increase in the efficiency and speed of the reaction, as well as an improvement in the selectivity and regioselectivity of the process, while reducing the reaction temperature [41].

## 3. ILs Suitable for Starch Dissolution and Gelatinization

**Figure 3:**
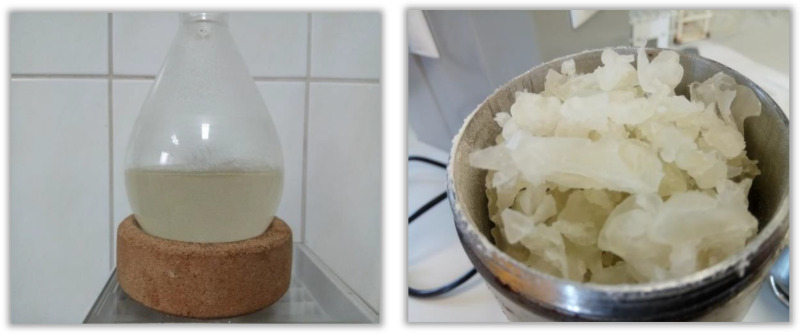
Starch gelatinized in 1-butyl-3-methylimidazolium chloride (wet and pre-dried form; Jan Dlugosz University, Czestochowa 2020).

**Figure 4:**
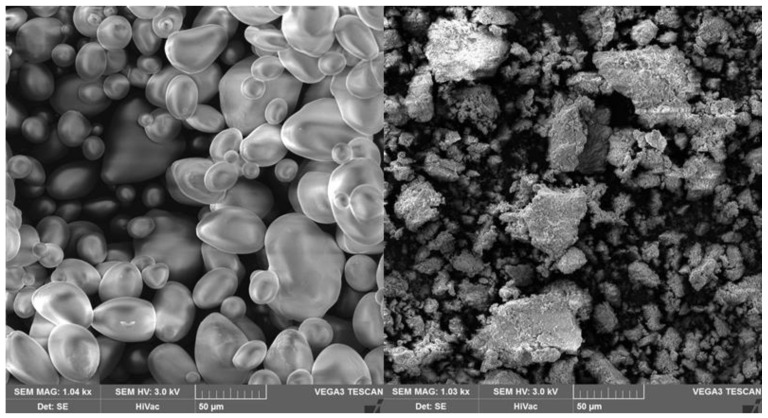
Destruction of potato starch grains after gelatinization in [BMIM]Cl (Jan Dlugosz University, Czestochowa 2020).

**Figure 5:**
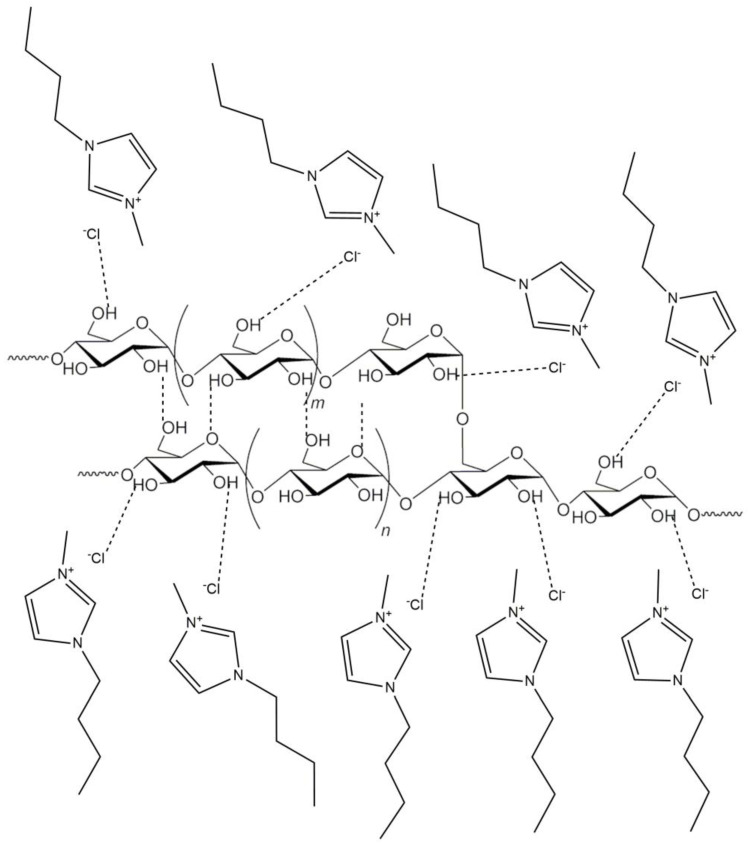
Mechanism of starch gelatinization in 1-butyl-3-methylimidazolium chloride.

The gelatinization mechanism of carbohydrate polymers, when using both classic hydrophilic organic compounds and hydrophilic ILs, is based on breaking intramolecular and intermolecular hydrogen bonds [14,42]. Of the ILs used for starch and its derivatives so far, simple liquids based on halide anions and imidazole cations with a short aliphatic substituent are the most common. In particular, 1-butyl-3-methylimidazolium chloride [BMIM]Cl and 1-allyl-3-methylimidazolium chloride [AMIM]Cl were the predominant ILs used as solvents and reaction media for starch [17,18]. Starch gelatinized in [BMIM]Cl and the destruction of its grains are shown in Figure 3 and Figure 4, respectively. When imidazole chloride ILs are used, new hydrogen bonds are formed, mainly between the anions or, to a lesser extent, between the imidazole cation and the proton of the polysaccharide hydroxyl group during gelatinization (Figure 5). Dissolution efficiency and other properties of an IL are closely related to its structure [43,44]. An IL composed of a cation with a small number of substituents and a short aliphatic chain along with a simple anion of a strongly electronegative element shows greater polarity than other ILs and thus a greater ability to dissolve hydrophilic compounds like starch [45]. The high polarity of the compounds used in the modification of starches or other biopolymers is a desirable feature when dissolving or gelling them but undesirable in anhydrous and biocatalyzed reactions. The hygroscopicity of the solvents causes problems, with the removal of water being a byproduct of the reaction. Moreover, this may be the main reason for enzyme inactivation through interactions with hydrogen bonds of the catalytic triad of amino acids and conformational changes within the active centers of enzymes [46].

Solvation of anionic nucleophiles is weaker in polar aprotic solvents than in polar protic solvents. The lack of hydrogen bonds between the nucleophile and solvent in polar aprotic solvents increases the nucleophilic character of the reagent. The solvation of cations is mainly favored by polar aprotic solvents since they stabilize the carbocation. Hence, it seems reasonable to use a simple aprotic IL with a short aliphatic substituent as a reaction medium in the synthesis of carbonyl starch derivatives, such as esters [47,48].

## 4. ILs as Starch Solvents, Compatibilizers, and Plasticizers

**Figure 6:**
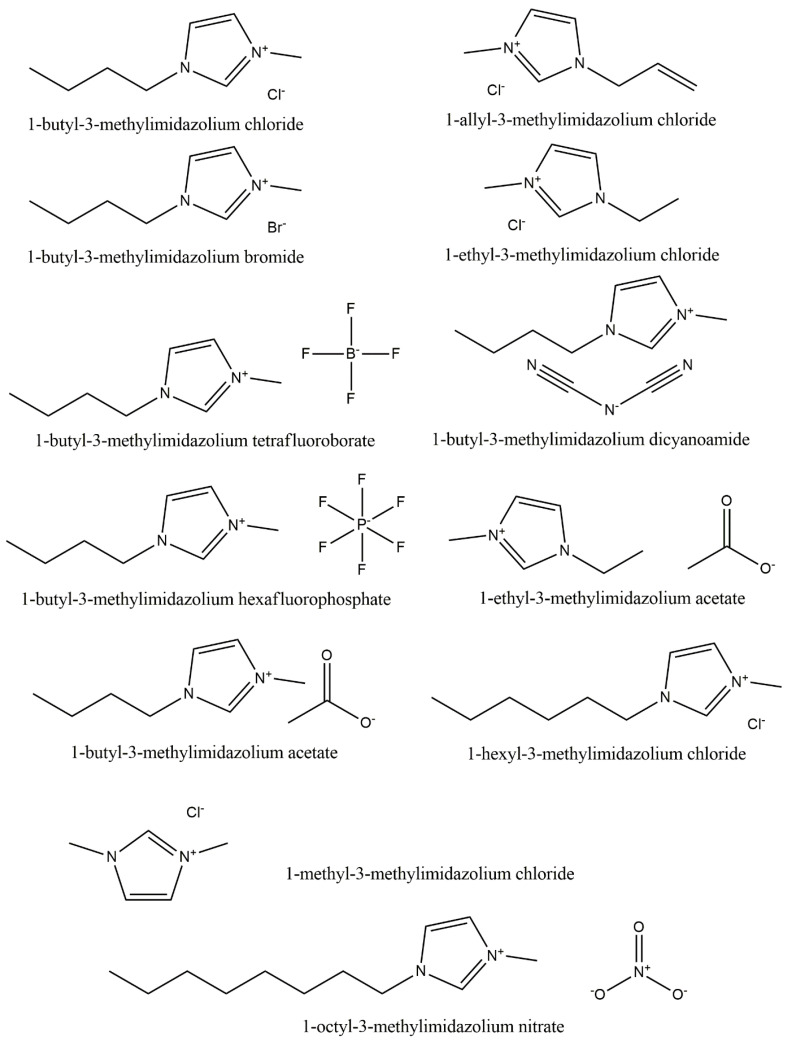
Structural formulas of the ILs most often used for the processing of starch-based materials.

The most commonly used ILs for the purposes of dissolving [42,49,50,51,52,53,54,55,56,57,58] and plasticizing [59,60,61,62,63,64,65,66] are 1-butyl-3-methylimidazolium chloride [BMIM]Cl, 1-allyl-3-methylimidazolium chloride [AMIM]Cl, and 1-ethyl-3-methylimidazolium acetate [EMIM]Ac (Table 2) [42,49,50,51,52,53,54,55,56,57,58,59,60,61,62,63,64,65,66]. The structural formulas of these and other popular ILs for the processing of starch-based materials are shown in Figure 6.

The effect of [BMIM]Cl on the dissolution and degradation of starch for its modification was studied by Kärkkäinen et al. [52]. The dissolution and degradation of six native starches (wheat, barley, potato, rice, corn, and waxy corn) under controlled microwave conditions and in an oil bath (conventional heating) were investigated by using the HPLC-ELSD method. The results suggest that [BMIM]Cl may indirectly play the role of the catalyst in starch depolymerization. Microwave heating of starch at 80 °C accelerated the dissolution and degradation process when compared to conventional heating in an oil bath at 100 °C. The authors noticed the immediate degradation of amylopectin after directly immersing starch granules in [BMIM]Cl, while degradation of amylose started later. Notably, the degradation rate of amylopectin depended on its origin and potato starch degraded slower than cereal starches. Hence, due to the degradation impact of this IL on starch, it cannot be used for the solvation of the starch species. The effect of several ILs on the dissolution and depolymerization of barley starch was studied by Lappalainen et al. [42]. The authors examined 10 ILs (1-allyl-3-methylimidazolium chloride ([AMIM]Cl), 1-butyl-3-methylimidazolium chloride ([BMIM]Cl), 1-hexyl-3-methylimidazolium chloride ([HexMIM]Cl), 1-butyl-3-methylimidazolium bromide ([BMIM]Br), 1-hexyl-3-methylimidazolium bromide ([HexMIM]Br), 1-methylimidazolium formate ([HMIM][HCOO]), 1-butylimidazolium formate ([HBIM][HCOO]), imidazolium formate ([HIM][HCOO]), 2-hydroxy ethylammonium formate ([NH_3_CH_2_CH_2_OH][HCOO]), 1-ethyl-3-methylimidazolium dimethylphosphate ([EMIM]Me_2_PO_4_)) as solvents and *p*-TsOH as a catalyst. The process was carried out at 80 °C using microwave heating. The depolymerization extent and dissolution time of the starch were specific to each IL. The authors demonstrated that barley starch immersed in the tested ILs was dissolved and depolymerized into water-soluble oligomers (1000–2000 Da). The reaction time was shorter for ILs with a smaller cation (e.g., [BMIM]Cl and [AMIM]Cl). A larger cation can cause steric hindrance due to the longer alkyl chain in some ILs (e.g., [HexMIM]Cl) and slow down the reaction.

In other studies, [BMIM]Cl was also involved in the preparation of biocomposites with thermoplastic starch [56]. The pretreatment of oil palm frond fiber with ILs ([BMIM]Cl and [EMIM][dep]) resulted in biocomposites with activation energy values 10–125% higher than those of the composites obtained without using ILs over a conversion range of α = 0.1–0.6. The use of ILs for the pretreatment of agricultural lignocellulosic waste enabled researchers to obtain biocomposites with thermoplastic starch under environmentally friendly conditions.

ILs are also used for the preparation of thermoplastic starch (TPS). Sankri et al. used [BMIM]Cl to obtain TPS [59]. The IL proved to be a better plasticizer than glycerol, and the obtained product was characterized by the property of low water absorption. Mechanical tensile tests proved strong plasticization of starch under [BMIM]Cl. The Young’s modulus was found to decrease more significantly than the glycerol plasticized TPS, while the elongation at break increased by four times from 100% to 400%. The very low, rubbery Young’s modulus of plasticized TPS with [BMIM]Cl (0.5 MPa) suggested a considerable decrease in hydrogen bonds between the starch chains affected by the presence of the IL. Further, Leroy et al. confirmed that [BMIM]Cl has better plasticizing properties for corn starch than glycerol [60]. The use of [BMIM]Cl led to lower hygroscopicity than the use of glycerol, resulting in the more efficient plasticization of both the starch and zein phases and compatibilization of starch/zein blends. Thus, this material may potentially be used to produce biocomposites from raw natural polymers and applied in food packaging and other areas. The plasticizing properties of [BMIM]Cl were also investigated for high amylose rice starch by Devi et al. [64]. The sol–gel phase transition temperature of native starch and starch with IL varied between 53.99 and 39.7 °C for the former and between 49.50 and 40.6 °C for the latter. The presence of [BMIM]Cl influenced the textural and electrical properties of the gel and improved its thermal stability. Bendaoud et al. also demonstrated that the ILs ([BMIM]Cl, [AMIM]Cl) are characterized by lower water absorption, being better plasticizers than glycerol. Moreover, 1-allyl-3-methylimidazolium chloride is a more efficient depressor of glass transition temperature than glycerol [66]. The crystal structure of maize starch measured by X-ray diffraction analysis (XRD) was destroyed by treatment with more than 22.5 wt% [AMIM]Clin the mixture; therefore, it may be an efficient plasticizer up to this value. The effective plasticizing properties of [AMIM]Cl were demonstrated by Zdanowicz et al. [67]. They also confirmed that [AMIM]Cl is an effective solvent for starch at a temperature of 80 °C. The research included the analysis of rheological changes and morphology of thermoplasticized starch systems and solubility tests performed on 5 wt.% starch solutions in ionic liquids.

ILs, particularly [AMIM]Cl, can be used for grafting starch. In one study, 1-allyl-3-methylimidazolium chloride was found to be a promising solvent in the ring-opening graft polymerization of L-lactide onto starch chains [49]. The obtained grafted starch with poly(*l*-lactide) (starch-g-PLLA) demonstrated good adhesion between the two components, evidenced by SEM observations. Although the grafting efficiency of PLLA reached 30%, the chains of the grafted PLLA were relatively short, as calculated according to a standard curve created by the FTIR method. Due to the high dissolubility of starch and chemical inertness, [AMIM]Cl was employed as a reaction medium for the preparation of a starch-based macro-initiator using atom transfer radical polymerization (ATRP) [50]. The authors successfully synthesized starch macro-initiators with varying degrees of substitution in [AMIM]Cl by homogeneous esterification with 2-bromoisobutyryl bromide at room temperature without using any additional catalysts. Using this type of esterification, the graft density and graft ratio improved more significantly than during heterogeneous surface-initiated polymerization. Another IL that can be used for the grafting of starch is 1-ethyl-3-methylimidazolium acetate. [EMIM]OAc was found to be an effective solvent for starch, able to disperse starch granules to molecular level without derivatization. Men et al. synthesized a copolymer of starch highly grafted with polystyrene (starch-g-PS) by utilizing [EMIM]OAc as the solvent and potassium persulfate as the initiator [51]. The grafting percentages calculated by the FTIR calibration method increased to 114%. The polystyrene side chains were evenly distributed on the starch backbone of the obtained copolymer. ILs are widely used as solvents or catalysts in various kinds of polymerization (cationic polymerization, anionic polymerization, radical polymerization, coordination polymerization, condensation polymerization, enzymatic polymerization) due to their easy miscibility with polar and non-polar organic and inorganic solvents, catalysts (inorganic and organometallic compounds), monomers, and polymers [68]. Abdolmaleki et al. used ILs (pyrrolidinium bisulfate [H-NMP][HSO_4_], pyrrolidinium chloride [H-NMP][Cl], and morpholinium bisulfate [H-Mor][HSO_4_]) as acid catalysts to prepare biodegradable polycaprolactone by ring-opening polymerization [69]. The resulting polymer had a good yield and inherent viscosity (0.10–0.18 dL/g). In the next step, the obtained polymer was used to improve the quality level and mechanical properties, as well as to reduce the hydrophilic properties of starch. The ring-opening polymerization of ε-caprolactone was tested in the presence of starch hydroxyl groups as initiators and ionic liquid as catalyst. The obtained starch-grafted polycaprolactone was characterized by 1H-NMR, FT-IR spectra, and field emission scanning electron microscopy analysis.

Lu et al. examined [EMIM]OAc as a solvent for maize and potato starch [53]. They investigated the effect of [EMIM]OAc/H_2_O ratio on the behaviors of both these starch types. The effects of the IL were found to be dependent on the starch source and structure. Gelatinization and dissolution of starch occurred competitively and synergistically when the [EMIM]OAc/H_2_O ratio was increased. Differential scanning calorimetry (DSC) results showed changes in the transition of starch from a single endotherm to an exotherm/endotherm and then to a single exotherm with the increase in IL. Excellent solvent properties of maize starch were confirmed by its low gelatinization temperature, which was only 44.4 °C in [EMIM]OAc/H_2_O. In another study, [EMIM]OAc was used as the solvent for a corn starch and cellulose mixture [55]. The authors found that even a high polymer concentration (up to 10 wt%) did not cause phase separation in the mixture. FTIR analysis of dry films confirmed the absence of new bonds formed between the components, and XRD proved a meaningful decrease in crystallinity. Liu et al. highlighted the role of coagulation in starch modification and compared its effects in ethanol and water. When coagulated in EtOH, starch was trapped in three-dimensional cellulose networks, whereas coagulation in water caused partial leaching of the starch, resulting in pores and channels. The morphology of the freeze-dried wet films demonstrated that pore size can be tuned by varying the mixture composition and altering the coagulation bath.

In recent studies, the effectiveness of [EMIM]OAc as a plasticizer in the preparation of starch-based films has been tested [61,62,70]. The authors compared the effect of this IL with glycerol and proved that [EMIM]OAc can damage the crystalline structure of starch more successfully and increase the mobility of the amorphous region via replacing starch–starch interactions with stronger starch–[EMIM]OAc interactions. Consequently, when plasticized with this IL, the obtained TPS film exhibited lower tensile strength and stiffness but higher flexibility. Moreover, this film showed better anti-aging effects and greater biological stability than films obtained using glycerol as a plasticizer. This is a great advantage from a mechanical and processing standpoint. Zhang et al. demonstrated that 1-ethyl-3-methylimidazolium acetate ([EMIM]OAc) may successfully modify starch into optically transparent electroconductive films [63]. The addition of this IL significantly reduced the processing temperature by compression molding at a relatively mild temperature (ranging between 55 and 65 °C), much lower than those commonly used in biopolymer melt processing (mostly over 150 °C). The mechanical properties of the films may depend on the starch crystallinity. The A-type crystalline structure of the starch was disrupted using the following postprocessing conditions: a higher [EMIM]OAc content, a lower processing temperature (55 °C), or higher relative humidity (75%).

## 5. ILs for Conventional Synthesis of Starch Ethers and Carbonyl Derivatives

In the case of the synthesis of starch carbonyl derivatives in ILs, corn, potato, and cassava starches have primarily been described. Only a few successful attempts to etherify starch are currently known. One of them involved the use of [BMIM]Cl in the reaction of corn starch with glycidyltrimethyl ammonium chloride (GTAC), catalyzed by sodium hydroxide [57]. The product of this reaction, which was conducted in the most optimal conditions (temp. 80 °C, 2 h), was cationic starch with a maximum degree of substitution (DS) close to 1. The studies showed that the excessive use of hydroxide as a catalyst led to the hydrolysis of GTAC, which was manifested by a decrease in the DS of the obtained products. Additionally, the possibility of recycling [BMIM]Cl, which did not significantly reduce the reaction efficiency, was confirmed. The ability to repeatedly synthesize a cationic starch derivative was explained by the homogeneous nature of the reaction system. Effective relaxation of the macrostructure of granular starch and access to its hydroxyl groups confirmed the effectiveness of the IL used. Cationic starch was also obtained by Lobregas et al. by grafting another IL—1-glycidyl-3-methylimidazolium chloride (GMIC)—onto the polysaccharide chain of potato starch [65]. It is also possible to obtain anionic maize starch with the use of ILs [54]. Therefore, a reaction between the polysaccharide and sodium monochloroacetate (SMCA) was performed. The highest DS (0.76) was obtained with synthesis carried out at 90 °C for 3 h. In this case, sodium hydroxide was used as the catalyst and [BMIM]Cl as the reaction medium. Thus, an IL based on simple imidazolium chloride has proven to be a suitable solvent for the synthesis of anionic as well as cationic starch derivatives.

The application of ILs in the oxidation of starch is not common [70]. Only polyoxometalate-based ILs (POMs) have been used as catalysts for starch oxidation. POMs are synthesized by a precipitation/ion exchange method with choline chloride and (H_5_PMo_10_V_2_O_40_) as precursors. A combination of POMs with choline chloride exhibited the same level of effectiveness as traditional catalysts such as Fe_3_SO_4_. Moreover, an IL-based catalyst for starch oxidation (such as POMs) has been found to be thermoregulated and ecofriendly.

The use of ILs in the esterification of starch with acid chlorides and anhydrides as well as short and long-chain carboxylic acids is much more common and has attracted great interest (Table 3). The effect of synthesized starch esters—hydrophobic derivatives—is more desirable due to its better utility and processing properties than native starch. One such esterification, which was described in the literature, involved the synthesis of starch acetates and succinates using [BMIM]Cl as the solvent [71]. Corn starch esters were prepared by pyridine-catalyzed reactions with acetic anhydride or succinic anhydride. Depending on the applied conditions, starch acetates with DS of 0.004–2.35 and starch succinates with DS of 0.02–0.93 were obtained. The authors explained the significantly lower DS when using succinic anhydride as an esterifying agent by its lowered reactivity as a result of increased steric hindrance. In both cases, the semi-crystalline structure of starch was destroyed during its dissolution in IL, which was confirmed by electron microscopy and X-ray diffraction studies. Luo et al. also obtained corn starch acetates with similar DS from reaction with an acid anhydride in the same imidazolium chloride and under similar temperature conditions (75–115 °C) [72]. The difference, however, was that the esterification was carried out using a catalyst. The reaction was preceded by dissolving starch in an IL at 105 °C for 2 h. Using the anhydride in molar excess to the anhydroglucose unit (AGU) and carrying out the esterification at a temperature above 100 °C turned out to be optimal for achieving the best degree of starch modification. The recorded diffractograms confirmed the formation of new semi-crystalline structures, and thermal analysis confirmed the increased thermal stability as a result of acetylation. In 2010, Volkert et al. used 1-butyl-3-methylimidazolium chloride and, more specifically, its salts (imidazole derivatives with carboxylic acid chloride) as catalysts for the esterification of corn starch with different amylose contents [73]. The role of the solvent was played by the acid anhydride or acyl chloride reactant. The presented method made it possible to obtain a mixture of starch esters (acetates, laurates) with a relatively high DS, i.e., above 2. The products showed improved mechanical properties and had more resistance to moisture than unmodified starch. In other studies, the same authors successfully attempted the esterification of high amylose corn starch in ILs [74]. Acetic or propionic anhydride served as the solvent, reaction medium, and esterifying agent. This is another example of ILs—mainly imidazolium chlorides such as 1-ethyl-3-methylimidazolium, 1-butyl-3-methylimidazolium, 1-methylimidazolium, or 1-hexylimidazolium—being used as esterification catalysts. By testing the catalytic properties of the above-mentioned ILs, the influence of their structure on the degree of starch degradation was analyzed. Thus, a decrease in the DS was observed with an increase in the aliphatic chain of the imidazolium cation. Based on the obtained results, the highest efficiency of starch esterification was achieved by catalyzing the reaction with 1-methylimidazolium chloride. The products of enzymatic hydrolysis of starch—maltodextrins—were also subjected to esterification in ILs. Corn maltodextrin acetates were synthesized by Shogren et al. [75]. This time, transesterification was carried out using vinyl acetate and 1-butyl-3-methylimidazole salt. The maximum DS of maltodextrin equal to 1.8 was obtained under fairly mild conditions, i.e., 40 °C for 70 h. The differences in DS were closely correlated with the presence of a specific counter anion of the imidazolium salt. The transesterification was observed to be more efficient when using ILs with alkaline anions than with neutral anions. In addition, due to the lack of significant differences in the molecular weight of maltodextrin, it was argued that there was no significant degradation of the polymer chain. The same research group performed transesterification of maltodextrin in a non-catalyzed reaction with vinyl stearate in 1-butyl-3-methylimidazolium dicyanamide ([BMIM]DCA) [76]. The reaction was carried out at 75 °C for 69 h and obtained maltodextrin stearates showing weaker or stronger hydrophobic properties, depending on the degree of modification. Gao et al. undertook esterification of maize starch with higher carboxylic acids (stearic, palmitic, and lauric) in classic organic solvents such as dimethyl sulfoxide (DMSO) and dimethylformamide (DMF) and ILs such as [BMIM]Cl and [EMIM]OAc and their mixtures [77]. It was observed that the longer the alkyl chain of the esterifying agent, the lower DS of the obtained product. However, in all cases, regardless of an acyl group donor used, low-substituted esters of starch and higher carboxylic acids were obtained. In 2011, Xie and Wang carried out the transesterification of corn starch with methyl esters of lauric and stearic acids [78]. Pyridine was used as the catalyst, and the reaction medium was well-known and widely used [BMIM]Cl. However, optimization of the reaction conditions did not allow them to obtain starch esters with a much higher degree of modification than in the previously presented studies. Microscopic and X-ray analyses confirmed the changes in the characteristics of starch esters. Interestingly, the authors found that even the fourfold recycling of the IL did not reduce the efficiency of starch transesterification.

## 6. ILs for Biocatalyzed Synthesis of Starch Esters

**Figure 7:**
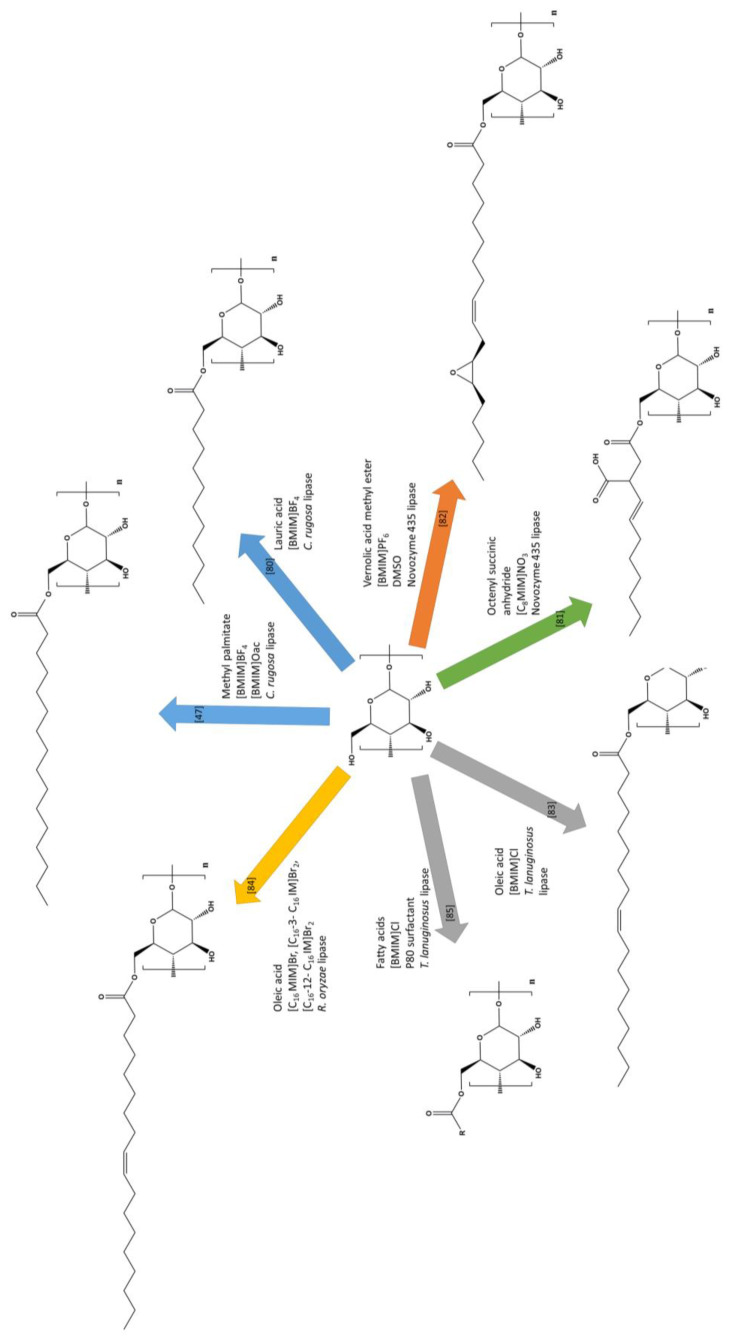
Biocatalyzed esterification of various types of starch (→ high-amylose maize starch, → cassava starch, → waxy maize starch, → potato starch, → corn starch) in ionic liquids.

Recently, the first successful attempts of enzyme-catalyzed synthesis of carbonyl starch derivatives in ILs were carried out (Table 4, Figure 7). One of the first reactions of this type was the transesterification of high amylose corn starch with methyl palmitate in mixtures of [BMIM]BF_4_ and [BMIM]OAc [47]. During experiments, it was noticed that although [BMIMO]Ac was a good solvent for the polysaccharide, it inhibited the activity of the biocatalyst used—lipase from *Candida rugosa*. The synthesized starch palmitates showed the characteristics of more hydrophobic but less thermally stable materials than native starch. Notably, the use of the enzyme did not result in a diametrically higher DS of starch esters than in classical methods. The maximum DS achieved with this method was only 0.153, and increasing the lipase concentration had no effect. On the other hand, lowering the reaction temperature and reducing the amount of byproducts gave hope for pro-ecological modifications. Over time, attempts to carry out esterification/transesterification of starch or its derivatives in two-stage reactions, preceded by pre-gelatinization, were made. For example, in 2013, lipase-catalyzed synthesis of high amylose maize starch laurates in the form of dried gel was carried out [80]. However, the gelatinization and reaction medium were not the same IL. [BMIM]Cl was used in the first stage and [BMIM]BF_4_ in the second. The experiment was carried out by the same research group that had previously undertaken one of the first successful trials of biocatalyzed synthesis of starch palmitates in IL. The reactions were catalyzed using the same lipase from *Candida rugosa*. However, due to the heterogeneous nature of the mixture, esters with the highest DS (0.17) were obtained after a slightly longer time (around 2 h). Other maize starch, namely waxy, was successfully esterified with octenylsuccinic anhydride in 1-octyl-3-methylimidazolium nitrate ([OMIM]NO_3_) [81]. This time, Novozyme 435 lipase was used as a biocatalyst, and the obtained starch esters were characterized by a very low DS (0.0006–0.013). In the preparation of octenyl succinates, the starch was first pre-gelatinized in [BMIM]Cl, and the appropriate reaction was carried out in the mentioned nitrate. Despite the use of various combinations of substrate and enzyme concentrations and various temperatures and times, high DS values were not achieved. Moreover, prolonging the reaction time had the opposite effect and did not favor starch esterification. This result was explained by the increased amount of water as a reaction byproduct, thus resulting in a shift in balance towards hydrolysis rather than synthesis. The XRD, SEM, and thermal analysis carried out during the study once again confirmed the effectiveness of the simple imidazolium salt as a gelatinization medium for starch, relaxing its macrostructure and enabling better contact between the reagents during esterification. The same lipase (Novozyme 435) was used by Desalegn et al. for transesterification between the methyl esters of vernolic acid (vernolic oil) and cassava starch [82]. The syntheses were conducted in the [BMIM]PF_6_ and DMSO co-solvent system, resulting in esters with a significant DS equal to 0.95. Similar results were obtained during the pyridine-catalyzed synthesis of cassava starch vernolates in [BMIM]Cl, but the reactions had to be carried out at much higher temperatures [79]. The epoxy starch esters obtained in both cases showed features of an amorphous material and were thus more susceptible to thermal degradation. Desalegn et al. obtained similar results from two different methods, thereby confirming the possibility of functionalization of cassava starch by enzymatically or chemically catalyzed transesterification in an IL [82]. Similar results with regard to the physicochemical properties of starch esterification products in IL were obtained by Zarski et al. [83]. This time, however, they were unsaturated potato starch esters. Polysaccharide was esterified with oleic acid in the reaction catalyzed by fungal lipase from *Thermomyces lanuginosus*. The enzyme immobilized on a polymer carrier was used to contribute to its stability and effectiveness. The IL pre-gelatinization method was used again, but the same 1-butyl-3-methylimidazolium chloride served both as the gelatinization and esterification medium. Such a procedure significantly simplified the method while maintaining a similar DS. The highest DS was obtained in the reaction conducted at 60 °C for 4 h (0.22). As in other studies on the biocatalyzed synthesis of carbonyl starch derivatives, the hydrolytic effect of water and the thermal inactivation of the enzyme with a temperature increase above the optimal value were emphasized. It was also noticed that the obtained starch oleates could be subjected to further functionalities due to the presence of unsaturated bonds in the aliphatic chain, e.g., by addition reaction. Adak and Banerjee esterified corn starch with oleic acid but in an environment of little-known ILs [84]. Lipase from *Rhizopus oryzae* was the catalyst, and microwave radiation was used as the heating source. Newly synthesized imidazolium ILs such as [C_16_MIM]Br, [C_16_-3-C_16_ IMBr_2_], and [C_16_-12-C_16_IM]Br_2_, which exhibited surfactant properties, were used in this reaction. The significant increase in the efficiency of biocatalyzed starch esterification and achievement of a high DS (2.75) was explained not only by the use of non-toxic ILs but also by more efficient microwave heating. Thus, the authors presented a highly efficient and environmentally friendly method of obtaining hydrophobic and thermoplastic starch derivatives in the presence of ILs. In 2019, Żarski et al. again used immobilized lipase from *T. lanuginosus* and [BMIM]Cl to esterify potato starch [85]. This time, hydrolysates of high-oleic vegetable oils (pure and waste rapeseed oil) were used as esterifying agents. However, the reactions were not carried out directly on potato starch gelatinized in IL but on dried gel. Hydrophobic starch derivatives with a much higher DS—above 1.2—were obtained under the most optimal conditions, regardless of the oil hydrolysate. The significant increase in the degree of esterification compared to previously developed methods [83] was explained by conducting the reaction in a three-component system, i.e., which consisted of hydrophilic IL, non-ionic surfactant, and hydrophobic fatty acids. It was found that the surfactant used—polyoxyethylene sorbitan monooleate (known as Polysorbate 80®; P80)—was of strategic importance in increasing the biocatalysis efficiency in the IL. It was pointed out that the addition of the small amounts to the reaction system prevented chloride recrystallization below 60 °C and thus enabled the syntheses to be performed at more optimal temperatures for lipase, i.e., 40–50 °C. It was also found that P80, due to its amphiphilic nature, increased the contact between the hydrophobic and hydrophilic phases. It was suggested that it also limited the access of [BMIM]Cl to the active center of the enzyme and, consequently, led to its inactivation. Moreover, a new and efficient method of potato starch gelatinization in imidazolium IL was presented in the mentioned studies. Until now, the maximum concentration of starch in gel with [BMIM]Cl ranged from 5 to 10%. It was proven by using SEM and X-ray methods that a reduced pressure of >20 hPa and temperature of 60 °C for 30 minutes allows for successfully obtaining up to 30% starch gel [85]. Potato starch esters obtained in ILs by Zarski et al. [85] have been structurally, physico-chemically [85], and functionally [86] characterized. Films extruded from them exhibited increased hydrophobicity and improved mechanical properties while retaining biodegradability and non-phytotoxicity.

## 7. Conclusions

The most commonly used ILs for the modification of starch to improve its properties are 1-butyl-3-methylimidazolium chloride [BMIM]Cl, 1-allyl-3-methylimidazolium chloride, and 1-ethyl-3-methylimidazolium acetate [EMIM]Ac. They are used in many applications, including the dissolution and gelatinization of starch. In addition, they are used as solvents in the ring-opening graft polymerization of the other polymers onto starch chains. Another process that successfully uses ILs is the preparation of thermoplastic starch, wherein they play the role of a plasticizer. ILs have better plasticizing properties for starch than the most popular plasticizer, which is glycerol. In particular, ILs are often used as solvents in the preparation of starch modified with acid anhydrides or short- and long-chain acids. The obtained modified starches have hydrophobic properties, which are more desirable in industries such as packaging. In recent years, there have been several reports on the preparation of hydrophobic starch derivatives by enzymatically catalyzed esterification with the use of ILs. ILs usually constitute the reaction medium or are used for the pretreatment of the starch (gelatinization). The starch esters obtained in biocatalyzed reactions in the presence of ILs are characterized by a high DS, which can be additionally increased by using non-ionic surfactants. Biodegradability, non-phytotoxicity, hydrophobicity, and improved mechanical properties are the features of films extruded from starch esters obtained in the enzymatically catalyzed reaction of starch with fatty acids such as oleic acid and hydrolysates of pure and waste rapeseed oil. The combination of biocatalysis and ILs is highly advantageous from an economic point of view (higher process efficiency), and it is also pro-ecological (shorter time and lower temperature of process).

## Figures and Tables

**Table 1:** A comparison of some properties of organic solvents and ionic liquids [19].

Properties	Organic Solvent	Ionic Liquids
Number of compounds	<1000	>1,000,000
Applicability	single function	multi-function
Catalytic properties	rare	common
Range of availability	limited	unlimited (“designer solvents”)
Vapor pressure	usually high	negligible
Flammability	usually flammable	usually non-flammable
Polarity	usually high	moderate
Cost	normal	expensive
Recyclability	rare	frequent
Chirality	rare	common, controlled
Solvation	usually weak	usually strong

**Table 2:** ILs for dissolving and plasticizing starch.

Ionic Liquid	Role	Application	Refrence
[AMIM]Cl	solvent	Grafting of corn starch with L-Lactide by ring-opening graft polymerization (ROP)	[49]
[AMIM]Cl	solvent	Grafting reaction of polystyrene (PS) and poly(methyl methacrylate) (PMMA) via radical polymerization (ATRP) using starch-based macroinitiator	[50]
[EMIM]Ac	solvent	Grafting of corn starch with polystyrene by the conventional free radical polymerization	[51]
[BMIM]Cl	solvent	Effect of [BMIM]Cl on dissolution and degradation of six different native starches (wheat, barley, potato, rice, corn, and waxy corn) under conventional oil bath and controlled microwave heating	[52]
[AMIM]Cl [BMIM]Cl [HexMIM]Cl [BMIM]Br [HexMIM]Br [HMIM]HCOO [HBIM]HCOO [HIM[HCOO [NH_3_CH_2_CH_2_OH]HCOO [EMIM]Me_2_PO_4_	solvent	Effect of ILs with various cations and anions on dissolution process of barley starch	[42]
[EMIM]Ac	solvent	Solvent effect on maize starch (MS) and potato starch (PS)	[53]
[BMIM]Cl	solvent	Homogenous carboxymethylation of corn starch	[54]
[EMIM]Ac	solvent	Preparation of starch-cellulose films	[55]
[BMIM]Cl [EMIM]dep	solvent	Pretreatment of oil palm frond fiber with ILs Preparation of biocomposites with TPS as a polymer matrix	[56]
[BMIM]Cl	solvent	Preparation of cationic corn starch by reacting corn starch with glycidyltrimethylammonium chloride utilizing 1-butyl-3-methylimidazolium chloride (BMIMCl) as a reaction medium	[57]
[AMIM]Cl [SBMIM]Cl	solvent	Optimization of the conditions for dissolution and depolymerization of potato starch industrial wastes in ILs. Dual role of SBMIM CL as a solvent and catalyst.	[58]
[BMIM]Cl	plasticizer	Plasticizer in the processing of (maize) starch	[59]
[BMIM]Cl	compatibilizer	Compatibilizer for the blends of starch and zein	[60]
[EMIM]Ac	plasticizer	Plasticizer of starch-based film preparation	[61,62]
[EMIM]Ac	plasticizer	Plasticizer in preparation of transparent conductive thermoplastic starch (TPS) at a relatively mild temperature (55 or 65 °C)	[63]
[BMIM]Cl	plasticizer	Plasticizer effect on sol–gel phase transition, rheological, and physical properties of high amylose rice starch	[64]
GMIC	plasticizer	Grafting 1-glycidyl-3- methylimidazolium chloride (GMIC) ionic liquid onto the polysaccharide chain to afford a cationic starch (CS)	[65]
[BMIM]Cl [EMIM]Ac [AMIM]Cl	plasticizer	Preparation of thermoplastic starch (TPS) with different ratios of ionic liquids	[66]

**Table 3:** ILs used for conventional esterification of starch.

Solvent/Medium	Substrate	Reagent	Catalyst	Product	DS_MAX_	Reference
**[BMIM]Cl**	Corn starch	Acetic or succinic anhydride	Pyridine	Starch acetate or succinate	2.35 (A) 0.93 (S)	[71]
**[BMIM]Cl**	Corn starch	Acetic anhydride	-	Starch acetate	2.11	[72]
**Anhydride** **Chloride**	Corn starch	Acid anhydride or Acyl chloride	Imidazolium based ILs	Starch mixed esters	~3	[73]
**Anhydride**	Corn starch	Acetic or propionic anhydride	[BMIM]Cl	Starch acetate or propionate	2.89 (A) 2.86 (P)	[74]
**[BMIM]X; X-halogen**	Corn maltodextrin	Vinyl acetate	-	Maltodextrin acetate	1.8	[75]
**[BMIM]DCA**	Maltodextrin	Vinyl stearate	-		2.4	[76]
**IL, DMF or DMSO**	Corn starch	Lauric, palmitic, or stearic acid	-	Starch laurate, palmitate, or stearate	0.105 (L) 0.098 (P) 0.092 (S)	[77]
**[BMIM]Cl**	Corn starch	Methyl laurate or stearate	Pyridine	Starch laurate or stearate	0.37 (L) 0.28 (S)	[78]
**[BMIM]Cl**	Cassava starch	Vernolic acid methyl ester	Pyridine	Starch vernolate	1.03	[79]

**Table 4:** ILs used for biocatalyzed esterification of starch.

Solvent/Medium	Substrate	Reagent	Catalyst	Product	DS_MAX_	Reference
**Mixture of** **[BMIM]BF_4_ [BMIM]OAc**	High-amylose maize starch	Methyl palmitate	*C. rugosa* lipase	Starch palmitate	0.153	[47]
**[BMIM]BF_4_**	High-amylose maize starch	Lauric acid	*C. rugosa* lipase	Starch laurate	0.171	[80]
**Mixture of** **[BMIM]PF_6_ DMSO**	Cassava starch	Vernolic acid methyl ester	Novozyme 435 lipase	Starch vernolate	0.95	[82]
**[C_8_MIM]NO_3_**	Waxy maize starch	Octenyl succinic anhydride	Novozyme 435 lipase	Starch octenyl succinate	0.013	[81]
**C_16_ MIMBr,** **C_16_-3- C_16_ IMBr_2_, C_16_-12- C_16_ IMBr_2_**	Corn starch	Oleic acid	*R. oryzae* lipase	Starch oleate	2.75	[84]
**[BMIM]Cl**	Potato starch	Oleic acid	*T.lanuginosus* lipase	Starch oleate	0.22	[83]
**[BMIM]Cl** **P80 surfactant**	Potato starch	Fatty acids	*T.lanuginosus* lipase	Starch esters	1.36	[85]
